# Slip Detection Strategies for Automatic Grasping in Prosthetic Hands

**DOI:** 10.3390/s23094433

**Published:** 2023-04-30

**Authors:** Peter Kyberd

**Affiliations:** Department of Ortho and MSK Science, University College London, London HA7 4LP, UK; p.kyberd@ucl.ac.uk

**Keywords:** slip, grip, sensors, slip detection, prosthetic hand, hierarchical control

## Abstract

The detection of an object slipping within the grasp of a prosthetic hand enables the hand to react to ensure the grasp is stable. The computer controller of a prosthetic hand needs to be able to unambiguously detect the slide from other signals. Slip can be detected from the surface vibrations made as the contact between object and terminal device shifts. A second method measures the changes in the normal and tangential forces between the object and the digits. After a review of the principles of how the signals are generated and the detection technologies are employed, this paper details the acoustic and force sensors used in versions of the Southampton Hand. Attention is given to the techniques used in the field. The performance of the Southampton tube sensor is explored. Different surfaces are slid past a sensor and the signals analysed. The resulting signals have low-frequency content. The signals are low pass filtered and the resulting processing results in a consistent response across a range of surfaces. These techniques are fast and not computationally intensive, which makes them practical for a device that is to be used daily in the field.

## 1. Slip Detection in Human Grasping

To hold an object stably, the forces of grip, friction, and gravity have to balance. If there is insufficient grip force, (when the vector of the grip force and the mass of the object is greater than the opposing force from friction), the object slides past the gripper’s digits. At this time, the normal contact forces will be reduced and the tangential forces increased [1].

The friction between the surfaces results from the surface roughness. The elastic distortion of the asperities on the surface create vibrations which relate to the surface texture, slide speed, and contact force [1]. For humans holding an object, their system uses both signals to detect the approach of slip, and its occurrence. Reflexes halt the slide [2,3]. From an engineering perspective, the slide is the error signal and it can be used to determine when to increase the grip force. It is of use in both robotics and exo-prosthetics. Research in detecting and responding to slip has been incorporated into prosthetic arms for some time [4]. Some authors have suggested it is a great deal more recent in robotics [5].

When approaching a grasp, humans can use their prior experience of the object to determine the correct force [3]. The presence of prior knowledge can be used in some forms of robot manipulation (where the characteristics of the target objects can be known and controlled). However, when operating in an unstructured environment this is not possible and the manipulator has to be able to react to the changing circumstances quickly. A prosthetic hand can only operate this way.

Work looking at incipient slip detection in humans and machines, suggest that a useful measure of the friction (and the required holding force) can be detected from the change in the shape of the contact patch between the fingers and the object [6,7]. The human system uses a large number of sensors in the digits tips (a density of over 1000 per square centimetre [8]), thus it can rely on detecting the change in contact area. However, the frictional vibrations are also used to trigger grip reflexes [9]. The natural response is faster than 110 ms [10], with adjustments to the forces made between 60 and 80 ms [2]. To create a stable grip, the human Central Nervous System (CNS) also imposes a safety margin of between 10 and 40% [11].

## 2. Prosthetic Gripping

The engineering requirements for a successful prosthetic arm are significant. To be useful, it has to operate in the field for long periods of time without adjustment or servicing. The user should not be inconvenienced by its presence and the prosthesis must be easy to use day to day. The device has to be compact enough to fit within the space of the joint (they cannot have actuators more proximal to the joint like the natural limb). The prosthesis has to be as low in mass as possible. New users are rarely prepared to expend as much time as an infant does in learning to control its limbs, so control has to be easily and quickly learned. If the device is powered, the user has to carry the power source all the time it is in use. While battery power is significantly better than it was previously, anecdotally, users are unwilling to recharge their batteries often. This means the power consumption still has to be as low as practical. To achieve this means the digital processing requirement should be kept modest [12,13]. Additionally, if the prosthesis is to be sensate, sensors must work within the envelope of the limb.

A prosthesis mechanism has generally been enclosed in an outer glove which protects it from damage and ingress of contaminants into the mechanism, this is not *as* essential to users as previously. There is now a new generation of mechanisms that do not need a glove for appearance or protection. However, the appearance remains important to some individuals, so some will wish the mechanism to work within a glove. Any product should not limit its market unduly, so any mechanism and sensor combination must also work within the glove. Unfortunately, the mechanical properties of the glove impact on the performance of the device and sensors [14]. Additionally, aesthetic considerations go beyond the glove and digit tips oversized by the inclusion of sensors are not likely be accepted widely.

It is common to wish for haptic feedback for prosthetic limbs [15], it may increase the ownership of the limb. However, from a control perspective there is little reason to believe it will be successful. The control chain for an externally powered hand is very slow. If the input is myoelectric (with a delay of more than 100 ms), the mechanism introduces a further delay of up to 400 ms [16], therefore to require a reaction to a signal that the object is slipping and then a response via the Electromyogram (EMG) would create a delay of as much as a second. When Okamoto et al. gave a feedback signal to operators they recorded a delay of over 200 ms before an EMG reaction [17]. As indicated, the natural solution is significantly faster.

Other complications related to prosthetic delivery include that the number of control channels from the user is restricted (currently limited to controlling one joint at a time, even with EMG pattern recognition control) so a person with a powered elbow and hand will be unable to control the grip force themselves while operating the elbow; therefore, a built in reflex to detect object slip is potentially useful [12].

### Slip Detection and Reaction

The process of controlling slip is in three stages: the first is to unambiguously detect when the slip has occurred, (and not to be confused with other external or internal signals). The second is assess how much slip has taken place and the third is to react in proportion to the slide. This is important for prosthetics as the hand should not automatically grasp as hard as it can, natural hands only perform gasps as gently as necessary [11]. This not only protects the objects, but reduces the effort to hold an object and so extend the time before the person becomes tired.

The earliest recorded attempt to detect a slip in a prosthesis was by Salisbury [4]. At the time, the number of channels available to feed information back to the operator were very restricted, thus it was clearly effective to keep grip force control entirely within the prosthetic system. An automatic reflex was created by using a piezoelectric crystal to detect the vibration in the surface of the hand when the object slid. This signal was amplified and the length of slide recorded with a Schmitt trigger (see Section 5). It was reported that the device was capable of detecting slip, but the addition of an external glove reduced the sensitivity by 20 db. This is a common problem for some prosthetic devices, they cannot work within a glove [18].

The challenge of detecting slip has resulted in a number of different solutions. Simple measurement can be achieved using Force-Sensitive Resistors (FSRs), an ink-based system where pressure changes the contact resistance [19,20,21,22]. This product is produced by various manufacturers, two leading ones are Interlink Electronics, 1 Jenner, Suite 200, Irvine, CA 92618 USA and Tekscan, 333 Providence Highway, Norwood, MA 02062, USA. Similar changes in force can be recorded using film-based sensors [23,24].

Friction-based vibration can be detected with microphones [25] and accelerometers [26]. Optical methods include distortion of a transparent rubber dome on the digit end. This can give an image of the changing contact patch [6,7].

One of the most advanced research device that has been used by more than just its original creators is the BioTac sensor [27,28,29]. It uses a conducting liquid within a digit tip and an array of electrodes to measure the change in impedance caused by localised changes in pressure. It is currently too large for field deployment and the restrictions on processing speed and size of processor still limit its use.

A second novel optical sensor is based around a flexible membrane with rod-like asperities on the inside surface. An external force on the membrane causes the surface to flex and the asperities amplify this motion. This change can then be imaged by a miniature internal camera and slip can be seen as a flow field in the movement of the ends of the asperities. This is the TacTip [30,31]. It has been used to allow robots to explore a tactile field [32]. It demonstrates that the practical use of a camera internal to an integrated sensor is no longer limited by the physical dimensions of the camera. A simpler optical sensor uses a photoelastomer to detect force. Stress on the elastomer changes the polarisation of light in proportion to the stress [33]. Both of these sensors have the ability to sample across a large portion of the surface of the fingertip and do not need multiple individual sensors to scan the area. To sample the same area with point sensors requires many single sensors which creates an increase in complexity and a concomitant reduction in potential reliability.

## 3. The Southampton Hand

The natural hand is capable of a very wide range of grasp forms, it is reconfigurable into a range of grip shapes. These have been classified in to a very wide range of different grasp types and subtypes [34,35]. While this level of analysis is detailed and captures the subtleties of grasping, it is less use to the designer of a prosthetic limb with fewer motions. It is better to use the limited classification of *Precision* and *Power* as characterised by Napier [36]. Even with a prosthesis capable of only two grasps, there is still a need to switch simply between the different grips. The control of the human arm is performed hierarchically with the top level of the Central Nervous System (CNS) performing strategic motions (push, pull, grab) and the lower levels coordinating between the grip shape and the grip force appropriate for the particular task and object. The CNS tends to maximise the contact area and minimise the contact force so that most grasps with the natural hand are below 12N [37]. The effortless control most humans have of their hands are the result of a considerable level of practice as the person was growing up and the wealth of practice they have when young. Prosthesis users are not prepared to devote as much time in learning to use a prosthesis.

The conventional way to instruct a prosthetic limb is the use of EMGs (electromyograms). These are the small voltages associated with muscle contraction. The natural solution uses many muscles to control the arm, and there have been many attempts to use machine learning to detect the intent [38,39], but these have many problems, not the least being the computation power to analyse the signals. In designing a multifunction hand the designer is faced with the challenge of making the operation as simple as possible. For early advanced hands the only option for the selection of a particular grip was limited to using the EMG input to switch through a list of possible degrees of freedom, which is slow. To circumvent this limitation, in the early 1960s Jim Nightingale suggested a hierarchical control format based on a model of the CNS [40]. Initial contact with the object dictated the appropriate grip shape and slip detection ensured the operator need not concentrate on balancing forces to maintain grasp. This concept has lead to a series of prosthetic systems; first laboratory-based [41], and ultimately used in the field [42,43], referred to as *The Southampton Hand*.

The concept is to control a multifunction prosthetic arm with simple instructions, for a hand that is two EMG channels, one to open and close the hand and the other to switch between control states. The hand opens in a precision grip and the degree of opening is proportional to the size of the EMG channel (usually a extensor muscle). As they relax the muscle, the hand closes on the object and the digits stop when they make light contact with the object. Depending on where the first contact is on the hand dictates the grip form, so digit tip contact results in a precision grip. Palmar contact instructs the hand to go into a power grip and contact on the side of the index finger tells the hand to close the fingers, swing the thumb round to the side, and close upon the side of the index finger.

At this point, the hand is gripping with the lightest touch so that the grip can be adjusted. Application of the second muscle causes the hand to go in to the grasping mode, which uses the slip sensors to increase the grip if the object slips. If the user wants to override this reflex they may do so with a second application of the other muscle (usually the flexor) then the reflex is turned off, but the hand can be instructed to increase the grip force by the user. Finally, the level of effort used to instruct the hand to open when holding an object can be made greater than an empty hand, making releasing the object more deliberate.

The overall progression of the Southampton Hand (and the parallel development of a whole arm prosthesis [44,45]) was to improve the different aspects of the mechanical design [46], sensors and sensor systems [25], and, finally, to use it as the first prosthetic limb to be controlled by a microprocessor in the field by a user [42]. Next, more controllers were added forming a bus-based system for the entire arm [47]. One key aspect for the hierarchical control is the detection and reaction to object slip, these investigations are the basis of this paper.

### Slip Detection in the Southampton Hand

There is a growing interest sensing contact between the device and the environment [48], which has resulted in different engineering responses. One is to build the sensors into the fingertips [49,50,51,52]. This can require modifications to the glove to allow them to work satisfactorily. Many prosthetic hands sense contact by detecting the motor stalling when the digits touch [13]. However, this provides no information about the forces distribution or slip. A different response is to build the sensor *into* the glove itself [18,53]. It is the presence of a glove which is also the reason why direct contact methods (such as track ball in the digit tips) are not used in prostheses.

In the development of the clinical Southampton Hand, the engineering constraints on the design of the mechanism ensured the electronic hardware was compact and with low power consumption. If the terminal device has curling digits with multiple joints wires running from fingertips sensors need to be kept to a minimum, this limited the number of sensors that can be practically employed. To achieve higher sensor density requires upstream processing and/or serial communications down the finger. The digital processing of the signals is also more demanding of power and although this problem is not as acute as it once was, user habits of not wanting to recharge their devices frequently mean it remains a driver to make practical systems as energy efficient as practical.

A few sensory systems have been built into clinically applied hands the Ottobock (Otto Bock HealthCare GmbH, Postfach 1260, Max Näder-Straße, 37115 Duderstadt, Germany), *SensorHand speed* has been using a SUVA sensor for many years, it detects shear between the thumb and the held object [20]. The Motion Control (Motion Control, Inc. 115 N Wright Brothers Drive, Salt Lake City, UT, USA.) FLAG function (Force Limiting Auto Grasp) uses multiple sensors in the fingertips to allow the grip force to be limited to sufficient to maintain a stable grip. While research groups have had limited field use of sensorised prosthetic hands [48,50,54], it is members of Nightingales’ group that have conducted the longest program of research into sensorised prosthetic hands in the field.

A similar method of slip detection to Salisbury used a stylus from a record player to detect the vibrations. It was employed by Codd [41,55]. This was combined with sensors on the palmar surface of the hand and within the hand mechanism so that there was closed loop control of position of digits (something not used in commercial hands until after 2010 [13]). The hierarchical command structure meant the user provided basic open and close instructions for the hand and an electronic controller (at first fixed logic, later computer controlled) determined the correct grip posture and grip force [25,40,56,57,58].

A concern with recording the acoustic slip signal using conventional microphones or styli, is that it is prone to interference from detecting airborne sounds, and the vibrations transmitted through the hand mechanism (such as from the action of the motors). This could be interpreted as a slip signal. Moore [25] attempted to decouple the microphone from the hand through mounting a microphone on a mass placed upon a rubber pad on the the distal end of the thumb, (the tip of the thumb that opposed the index finger was also roughening up). While successful in reducing the signal from the hand it was less effective at reducing airborne noise.

**Figure 1:**
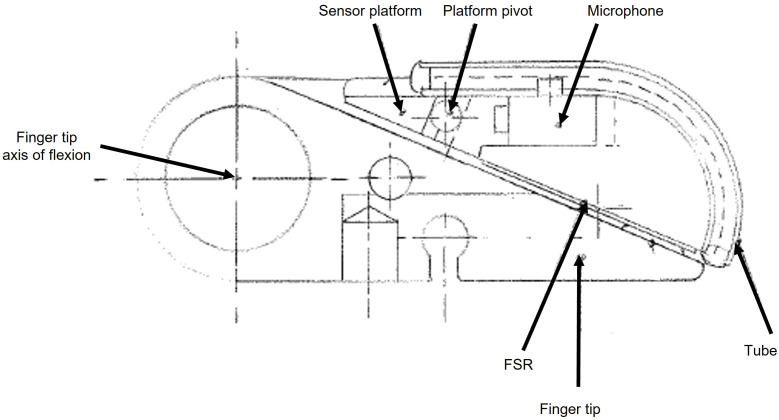
The Southampton tube sensor. The sensor is mounted on a platform on the digit tip which also acts upon the Force-Sensitive Resistor. The tube directly connects to the slipping object and so the frictional signal is coupled to the air in the tube and so the microphone. The airborne noises are poorly linked and so are much smaller and can be removed from the slip signal. (Design thanks to the late Mervyn Evans).

Barkhordar created a more effective means to separating signal from noise [57]. A hearing aid microphone (EA series microphones, Knowles Electronics Co. 73 Victoria Road, Burgess Hill, West Sussex, RH15 9LP, UK) was arranged with its stub-pipe through the wall of a rubber tube, which was then in contact with the grip surface, as shown in Figure 1. Slip signals vibrate the wall of the tube. The signals are readily transmitted to the air within the tube and onto a microphone. Airborne vibrations have to excite the rubber of the tube before they can create a signal in the tube. Hence, the impedance of the path is matched more closely to surface signals than airborne noises. This means the interference signals are much smaller, (micro-volt for interference compared to milli-volt for slip signal), and so interference can be thresholded out [57]. The tube can be any shape or size, curved or straight, allowing it to fit around the end of the finger. As the tube becomes larger or longer, then the surface becomes more like the membrane of a conventional microphone, hence for a longer tube the detected interference signal also became larger and the sensor becomes less able to discriminate [57]. This sensor has the advantage in that it can work *inside* a prosthetic glove [59] or built into the fabric of the cover [53]. An additional feature for this design is that if the tube is straight, it can incorporate a LED/photodiode pair at opposite ends. This beam can be used to measure force on the contact area as the light transmitted along the tube is proportional to the imposed force [57,60].

The key to transduction methods is to create a physical change that has a low energy loss and this transition is reversible and repeatable [58]. The collapse of the tube and the subsequent reduction in transmitted light achieves this [60].

In common with others, Nightingale’s team also used forms of elastomer as the spring material to translate movement from a load into a change in an electrical property. The property can be as part of the material itself, (i.e., compression causes the plane of polarisation to change [33]), or that a movement of a magnet placed on the top of the elastomer changes the local magnetic field of a Hall effect sensor [61]. A more sophisticated version is to embed the ferromagnetic material *into* the elastomer so that its compression increases the local density and so the magnetic field [62].

Early work by the team used a device based on the mutual induction between pairs of coils, one above and one below the foam. Force compressed the foam and changed the inductance between the two coils [63]). A later sensor employed a carbon-loaded foam over interdigitated contacts. Compression of the foam changed the bulk resistance which was detected between the two contact tracks [25]. A similar principle, but with the change being in the surface properties, is used later in Force-Sensitive Resistors (FSRs) [58] and later still a conductive cloth was used to make sensors built into the fabric of the glove [18]. The term *Force*-Sensitive Resistor is somewhat of a misnomer as both of these effects are area-based and, thus, are truly *pressure*-related. There are many similar technologies which have been used to detect slip through a shift in the pattern of forces across the surface of the hand [19,20,21,22,63,64].

## 4. Friction-Generated Slip Sounds

The detection of slip through measurement of the sound generated when two objects slide against each other clearly depends on the materials in contact and the nature of the slide. It has proved to be a useful means to detect slip. Anecdotally, some have suggested its usefulness is limited compared with the detection of the change of normal and tangential forces, because the signal only exists once the slide has taken place, so it is reactive not predictive. This is not supported by the experience from the natural solution [10], where the vibration is as important or more important than changes in force distribution. Additionally, it does not take into account that movement can occur at one point and not the entirety of the held object (so called “partial slip” [63]). Nor does this critique take into account that the movement of the hand holding the object can cause changes in the tangential forces without any slide (such as wrist rotation) so that there will be a false detection of slide with this method too. For a practical prosthesis it is important that whatever means is used to detect slip is consistent and reliable. For acoustic slip detection this means to ensure the hand has a consistent response to slips irrespective of the object being held or the tension of the grip. To ensure this, an understanding of the source of the slip signals is required.

### Vibrations from Frictional Contact

The sounds generated by objects sliding past each other are created by the friction between the surfaces. The surfaces are never truly smooth but with random asperities over them. The primary source of the signals are due to the contact between these surface asperities. Their elastic deformation creates sound [1,65]. As a result, frictional sounds are rarely stationary or ergodic [1]. Friction increases with roughness and speed [66]. The processes to generate the signal are chaotic in nature [67]. This ensures that the signals are broadband noise signals. The structure surfaces mean the energy is partitioned equally across the frequencies [1]. The energy of a signal increases as the frequency increases, the amplitude of individual frequencies declines as the frequency increases. Similarly there is a square relationship between elastic energy release in the signal generation and load [68], which means the dominant frequencies for a slip signal are low and the power in the signal decreases quickly as the frequency climbs.

Sounds generated due to sliding are broken down into two basic forms, “hums” and “squeals” [69,70]. For the hums the frequencies are broad with most below 500 Hz [71]. The squeals are mode-locked responses to the surface details [1] they are only generated from higher contact forces and sliding speeds. The definition of ‘higher’ depends on the surface. Smoother surfaces generate squeaks at lower speeds and pressures. For prosthetic slip, the interest is in detecting starting slides and in light grasps, thus the high-frequency conditions do not occur and it is possible to focus on low frequencies and low contact forces. Earlier work by the author [60] on the design of a slip sensor based around a rubber tube showed that the design reduces the very broad bandwidth of the core signal to around 500Hz. The mean frequency was measured and was shown to relate to the slip speed, but be broadly independent of grip tension. Similarly, the detected signal is broadly independent of surface or grip force. This paper investigates these relationships further.

## 5. Detection Methods

The second stage of the response to slip is to derive a measure of the amount of slip that has occurred. This needs to be performed as efficiently as possible. Historically, some signal processing was performed using analogue external circuits, more was undertaken using digital signal-processing techniques. However, power consumption for the device is proportional to clock speed and so equivalent devices with lower clock speeds have a lower power consumption. Even today this consideration is important for users. While some publications report algorithms have a low power consumption, they do not supply an estimate for the required processing power, for example [72]. Its feasibility is thus difficult to gauge. Some of the processing methods reported relay on machine learning [19,22,28,29,51], which would seem to restrict their application at present.

In addition, recent research shows in greater detail the way a prosthetic device is used in the field. One important finding is that even powered prosthetic hands are used passively more than they are employed actively [73]. While the motor consumes greater power while the hand is opened and closed, the energy to drive the electronics is potentially a more important factor in battery life as it is being consumed the entire time a device is on. Methods to conserve power are possible, but no consideration of this is reported so far. Thus, even with modern electronics it is still important to reduce the clock speed of the microcontroller to preserve power as much as is practical [42]. Solutions such as the design of the sensor to reduce interference (rubber tube) and some input circuitry to filter the signal (see below) is acceptable.

### Pulse Counting

Salisbury [4] was followed by Codd [55], who used a Schmitt trigger on the acoustic signal to generate a series of pulses with widths proportional to the instantaneous frequency of the slip signal. The timing of sequential rising or falling edges is proportional to the frequency of the slip signal and its duration. Rising edges can then be counted. This is simpler but it discards some information about the underlying signal that could be captured by measuring the waveform length. This is computationally simpler than extracting the frequency from signal processing methods [13,74].

Others have also employed similar methods that use the vibration signal to trigger a grip reflex [21,75]. By contrast, instead of thresholding the signal, Cordella differentiated the signal to obtain the spike at the beginning of a slide [76] to detect slip.

## 6. Hand Response

The final stage is to determine the response of the hand to the slide. The natural solution applies the least amount of force required to hold an object, which reduces the effort required [2]. However, this reflex requires prior knowledge of the object and surface properties [3]. For a prosthesis, it is less possible to know a priori what are the properties of the target object, so a more reliable response, once a slide is detected, is to increase the grip force until the sliding is arrested. Moore [25] used the recorded pulse count as the demand to an analogue motor driver, and similar techniques feed the number to input of a digital Pulse Width Modulator (PWM) drive.

This ties the response of the device to the surface texture of the held object. Two aspects of the texture have an impact. The surface finish will trigger a signal related to the size of the features, giving higher frequency for features of a smaller dimension. However, the lower frequency signals are common to the majority of surfaces, so that if the signal is low pass and band limited then the detected signals are not effected by different surface textures. The other factor, the slip speed, does create a greater demand which is needed to cause the hand to react faster to a faster slide [64].

## 7. Materials and Methods

The experience of Nightingale’s group across many years of field use of the vibrotactile sensor shows the advantages and limitations of the method. The sensor used is based around the rubber tube. Its structure filters the external signals above 500 Hz, and it has been shown that filtered this way removes the dependence on slip speed and across the range of surface finishes [60].

This method is simple and straightforward to undertake, and detects slip easily. It provides good separation of the signal from potential interference, but as the grip becomes tighter the acoustic connection between the object and the hand can become sufficient that the reflex causes the hand to impart maximum force.

Initial work looked at the properties of the Southampton tube sensor to explore the limits of its performance [60]. Experience with the system being used in the field on the Leverhulme/Oxford Southampton Hand [59] showed that it was effective, but pointed to a few limitations that additional signal analysis could overcome. Three areas of concern were the false triggering of the slip signal when small movements created very short pulse trains, the inability to detect very smooth surfaces as these only generated short pulse trains that otherwise looked like interference or non-movement, and, finally, the tendency when the hand grips tightly for the coupling of the slip sensor to become too great. Responses to these concerns were developed.

**Figure 2:**
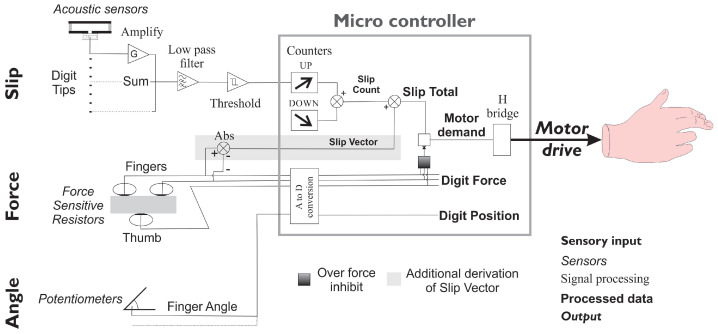
Schematic of Southampton Hand controller, including slip processor. Tube sensors detect the acoustic signal from the slip. The signals are filtered, added together, and then thresholded to generate a pulse train which is counted on input to the microcontroller. A second internal counter/timer counts down to remove short pulse trains (such as knocks and squeaks). An extra measure of slide, included as a result of this study, is derived from the difference of the force sensors on the fingertips which ensures that slips are detected and other interference signals are ignored (see Section 10).

The method is shown in Figure 2. The acoustic signal is amplified and passed into a Schmitt device, before the signals from three digit tips (index, third, and thumb) are input to the microcontroller (a PIC18F series (Microchip Technology Inc., 2355 West Chandler Blvd, Chandler, Arizona, USA), these features are widely available across the PIC range, the choice of specific device was based on other considerations).

Following some amplification, the three signal were combined and filtered before being thresholded by the Schmitt trigger. This signal triggered the PIC’s data capture interrupt automatically which increased an internal timer/counter. Short pulse trains were removed by using a second inbuilt timer to count *down* the pulse total. This count was reduced further if the grip force was sufficient to enhance the coupling between the hand and the object. As a result of this study, a further modification was to use the differential signals from the digits to inhibit/enable slip demand resulting from particular circumstances (see Section 10).

## 8. Method

The response of the slip system was tested using a similar set up to previously [60]. A series of standardised object blocks (wood, sawn and smooth paxilon, foam, glass bottle) were allowed to slide past a tube-based sensor mounted in the fingers of a prosthesis and the signal generated was analysed using Matlab (Mathworks, Natick, 1 Apple Hill Drive Natick, Massachusetts, USA), and the Fourier transform found of the signal was used to create power spectra. Additionally, the impact of the pulse train was studied. The acoustic signal was amplified and passed to an inverter. The data were obtained for the objects sliding past the sensor 10 cm four times (grip reflex not turned on). The raw count along with that after applying the down counter were recorded.

The analogue data were converted to a digital stream by the PIC at a rate of 2.5 KHz per channel. The slip count was the sum of the pulse input and the down counter (which is one of the internal timer/counter functions) decrements the count each time the timer reaches zero. The timer reset value is calibrated to give a decrement rate of 50 Hz so that short pulse trains are quickly reduced while longer ones add to the overall total (see Section 9.2 for impact of the process on the resulting count).

## 9. Results

**Figure 3:**
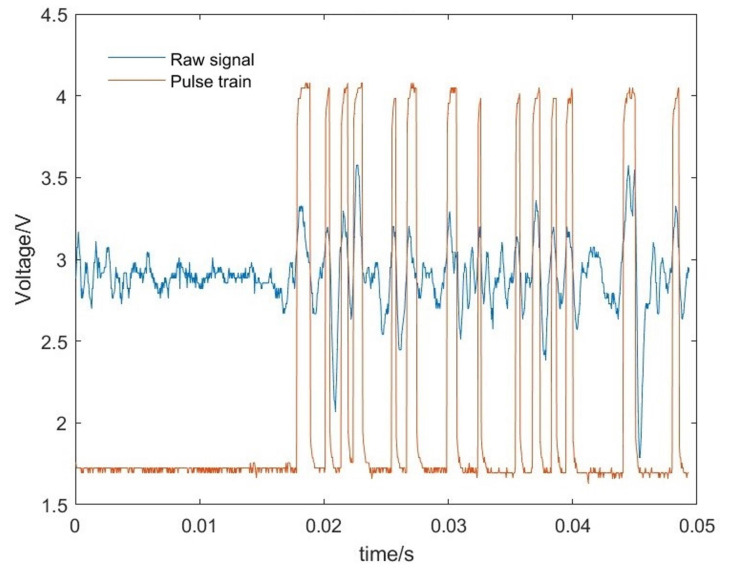
Slip signal and pulse train. The slip noise signal is superimposed upon the pulse train created by thresholding the filtered signal via a Schmitt trigger. It shows some noise from the hand mechanism being ignored. At 1.8 s the slip starts and 14 pulses are then generated.

An example of a typical trace overlaid with the pulse train generated by the trigger is Figure 3. In the first 1.8 s it shows some noise prior to the slip that was too small to be counted as a slip signal and 14 pulses subsequently as the sawn paxilon block sides past the sensor.

### 9.1. The Acoustic Signal

**Figure 4:**
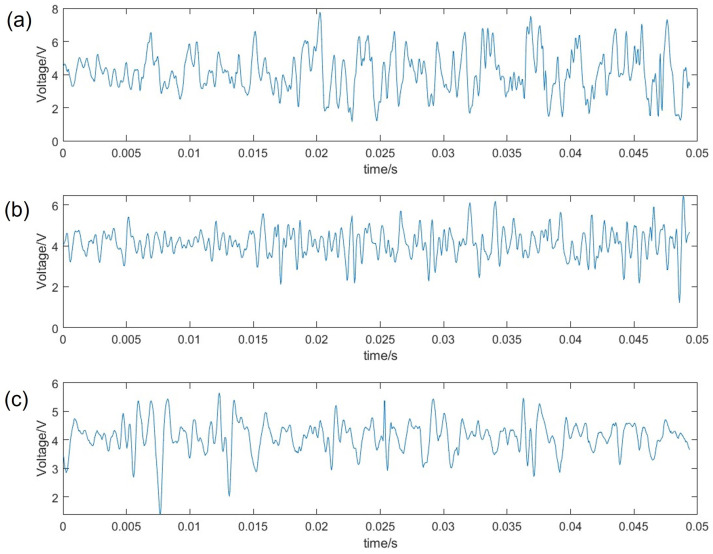
Acoustic slip signal for foam with changing tension. (**a**) 15N, (**b**) 10N, and (**c**) 5N.

The impact on grip tension on the frequency response is shown Figure 4. It reveals that there is greater power in the signal as the tension increases and the coupling between the object and the sensor increases; however the bandwidth remains the same with low-frequency components.

**Figure 5:**
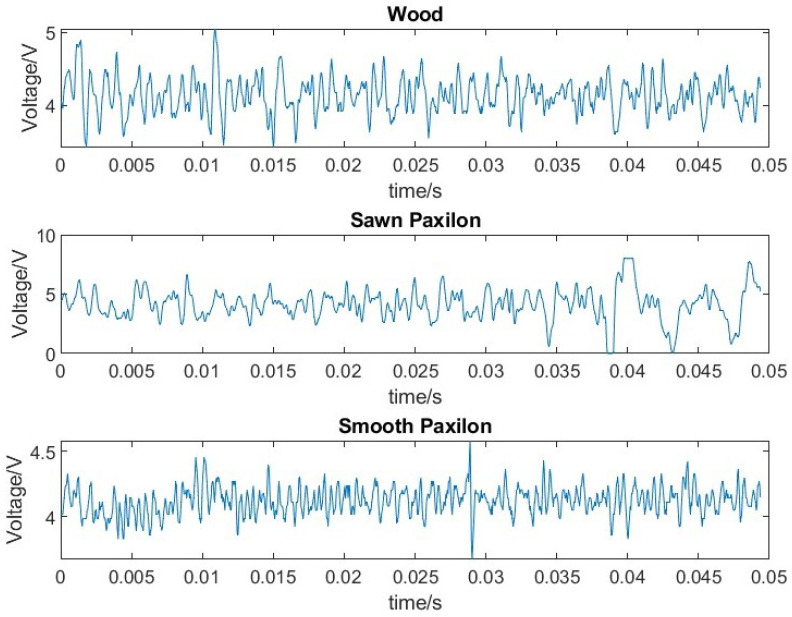
Acoustic slip signals for materials with different surfaces.

Figure 5 shows examples of different surface textures with the smoothest hard plastic (paxilon) generating lower energy signals. Smooth surfaces have smaller signals, with higher frequency content which is reduced by the sensor design and the filtering of the input signal.

**Figure 6:**
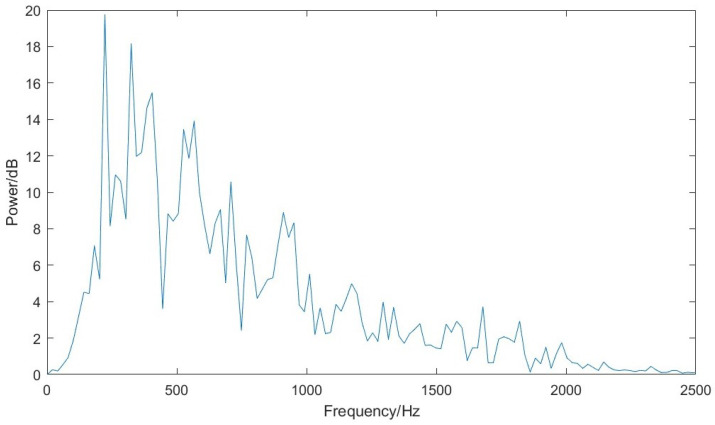
Mean power spectra for the acoustic slip signal from sawn paxilon for three slips. As the energy is evenly partitioned between frequency, the signal size will decrease with increasing frequency.

Figure 6 shows an example of the power density spectra for the mean of three runs for a single surface texture (Sawn Paxilon). This is typical of the different surfaces; more power at lower frequencies and diminishing power as the frequency increases.

**Figure 7:**
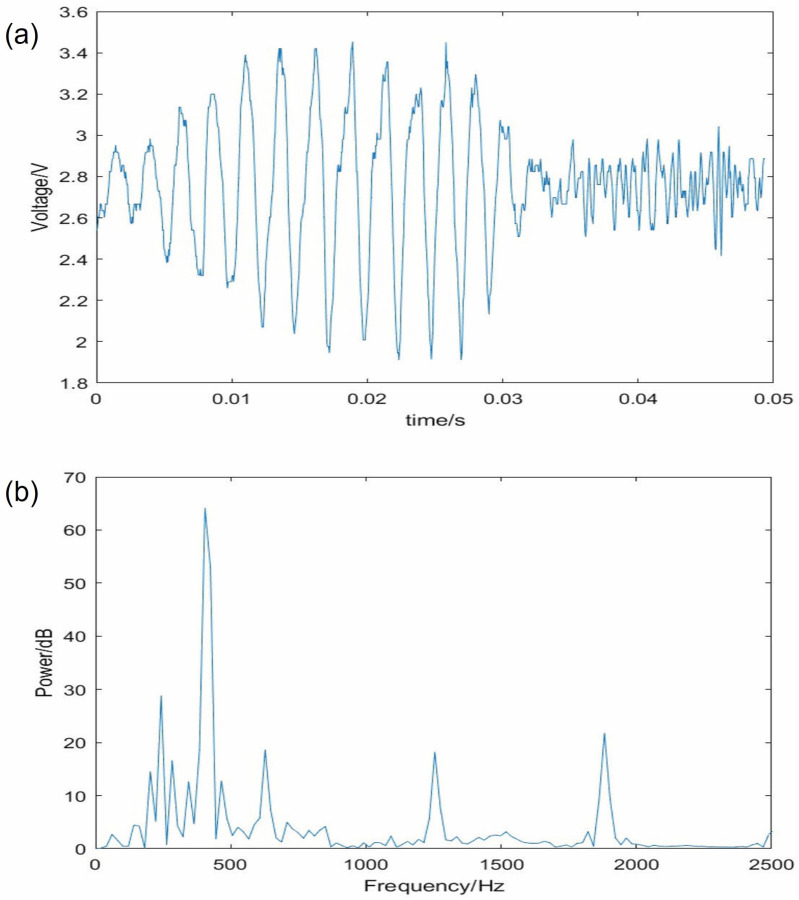
(**a**) Acoustic slip signal and (**b**) mean power spectra for a glass bottle. The peaks at intervals in (**b**) are suggestive of a mode-locked response to the sliding, which is indicated by the large regular peaks in (**a**).

Figure 7 shows an example of the signal for a smooth surface (a glass bottle) sliding past the sensor. The lower trace is the spectra for the mean of three runs. The early smooth signal would result in a count of 10 over 0.02 s. This is about 460 Hz and peaks at this frequency and harmonics are clear in the spectrum. This suggests the friction has produced mode-locked squeals on the smooth surface. Some squeaking was audible during the tests.

### 9.2. Pulse Counting

**Figure 8:**
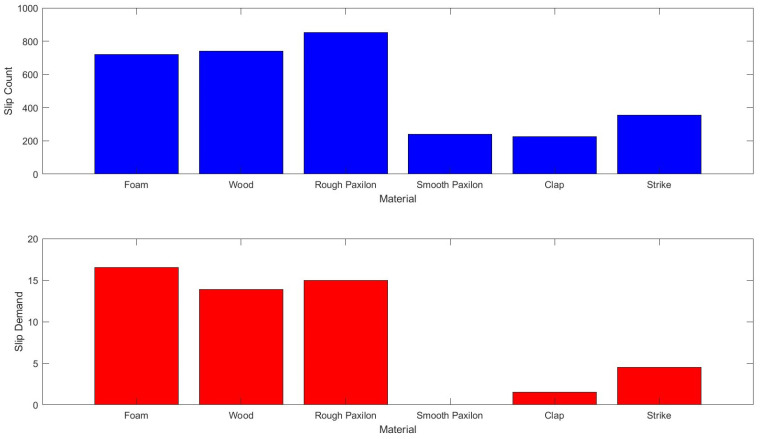
The slip count algorithm: the raw numerical count of the input pulses when the different materials are slid past the sensor for 10 cm are shown top. The interference from external noises and striking the hand are also shown. Bottom is the demand that results from the down counting. This final number is used to create the final demand that is used to drive the motor.

Figure 8 shows the raw number of counts for the different materials and the processed counts the totals for the processed counts are lower. Of note is that the processed counts for smooth paxilon are zero while those for taps and knocks are far lower than the other surfaces. In addition, the counts for different textures (some reasonably smooth, the wooden block) are similar to the rougher surfaces, denoting a consistent response. External signals (clap and strike) are measurably different from all other signals.

## 10. Discussion

Literature suggests that the energy generated by the friction of objects sliding past each other is equally partitioned between frequencies [1]. As the energy of a wave is related to its frequency, it is, therefore, to be expected that the amplitude of the signal should fall off rapidly as the frequency increases. The data from all the studies shows this tendency with the increased coupling creates a larger signal, only the smoothest surfaces generate squeaks rather than hums. The pulse generation alone separates signal from noise in all but the smoothest surfaces; however Figure 8 shows that the removal of short pulse sequences reduces the largest external noises, and removes the smooth surfaces. Therefore, *there is a difference* between the slip and the noise signals, even between the smooth surface and the interference. Based on this result, the slip vector calculation was added (the additional branch of the control diagram in the grey box).

While it is possible to derive the normal and tangential forces by a number of different measures, the addition of more sensors on the hand potentially increases the complexity and reduces the reliability, thus the three FSRs on the digit tips were used in combination to detect changes in the normal and tangential forces. The algorithm is a simplified version of the measure developed for the Southampton Hand [64]. It takes the difference between the two fingertip sensors and the thumb. If the grip was stable the difference would be static, as the mass shifts so does the vector between the hand and the object and this is detected. It can be considered as a measure of the imbalance between the opposing forces between fingers and thumb.

$$\begin{array}{c} Slipvector=Abs(\Delta (Digit1Force)-\Delta (Digit2Force)\\ Slipvector=Abs((ThumbForce-Finger1Force)-(ThumbForce-Finger2Force)) \end{array}$$

Alone, the force vector method has the potential to increase the grip when the hand is moved and the orientation of its wrist changes, while no slipping has occurred. To overcome this, the force vector slip signal was added to the vibrotactile slip signal count and a threshold applied. The slip response from the hand is only above a threshold when both heave and noise are occurring. This condition removes squeaks and short pulse trains from being interpreted as slip, so the hand reacts only when both slip signals are detected.

(1) Slipdemand=SlipVector+Slipcount

### Feedback to The User

An EMG signal is low frequency, below 1kHz. As Smit’s analysis of the control channels indicated [16], the delays introduced by EMG inputs from the user (more than 400 ms) are likely to be too slow to be useful in controlling the slide. However, at the same time, feeding the information that the object is stable or slipping, may well increase the users’ feeling of embodiment in the device, so simple sensors such as these also have a role in giving the user feedback.

## 11. Conclusions

Detection of the slip within the grasp of a prosthetic hand can be achieved using simple transducers which are designed to reduce the interference from other signals. Simplified, fast methods then process the signal. These methods do not need large amounts of processing power (which is not desired by the user population). Additionally, it is probable that internal reflexes to counter slip are necessary for prosthetic limbs as the delays introduced by the command channel (EMG) and the mechanism are too slow to allow a user to be consciously in the loop, while the automatic reflex is much faster. The sensor has been used in field trials of prosthetic systems for many years. The derived slip signal is also available for feedback to the operator, should that be considered necessary.

## Data Availability

Not applicable.

## Abbreviations

The following abbreviations are used in this manuscript:

CNS	Central Nervous System
EMG	Electromyogram
FLAG	Force Limiting Auto Grasp (Motion Control product)
FSR	Force Sensitive Resistor
LED	Light Emitting Diode
LOSH	Leverhulme Oxford Southampton Hand
MARCUS	Manipulation And Reaction Control under User Supervision
PIC	Programmable Intelligent Computer or Programmable Intelligent Computer
	—A Microchip product
PWM	Pulse Width Modulator
SUVA	Schweizerischen Unfall-Versicherungs-Anstalt, (Swiss Insurance Agency)
ToMPAW	Totally Modular Prosthetic Hand with A high Workability
